# Revisiting the psychology of waste: Replication and extensions Registered Report of Arkes (1996)

**DOI:** 10.1098/rsos.250367

**Published:** 2025-05-07

**Authors:** Zijin Zhu, Gilad Feldman

**Affiliations:** ^1^ Department of Psychology, The University of Hong Kong, Hong Kong

**Keywords:** wastefulness, avoidance, bias, judgement and decision-making, Registered Report, replication, sunk-cost effect, outcome bias

## Abstract

Arkes (Arkes 1996 *J. Behav. Decis. Mak.* **9**, 213–224. (doi:10.1002/(SICI)1099-0771(199609)9:3<213::AID-BDM230>3.0.CO;2-1) demonstrated a phenomenon of wastefulness avoidance, showing that people’s decisions are impacted by wastefulness, making decisions that avoid appearing wasteful. In a Registered Report with a Prolific sample (*N* = 659), we conducted a replication and extensions of studies 1, 2 and 3 from Arkes, 1996. We found empirical support for the impact of waste on evaluations of decisions in the movie package scenario in study 1 (original: *h* = 0.43 [0.03, 0.83]; replication: *h* = 0.26 [0.10, 0.42]) and on hypothetical decisions in the tent project scenario in study 3 (original: *w* = 0.23 [0.00, 0.52]; replication: *w* = 0.09 [0.00, 0.17]), but with no support in the tax program scenario in study 2 (original: *w* = 0.27 [0.00, 0.55]; replication: *w* = 0.03 [0.00, 0.12]). Our extension employing a continuous willingness measure, to supplement the scenarios’ dichotomous choice, showed similar results. We added a manipulation check extension, which showed that the manipulation worked as expected in scenarios 1 and 3, but not in scenario 2. In our extension examining reasons, in the successfully replicated scenarios, we found that in scenario 1, utility maximization was not rated as the most important, and in scenario 3, minimizing waste was rated as the most important reason. Overall, we concluded a mixed replication, with a successful replication of two of the three tested studies. Materials, data and code are available at https://osf.io/gf8rc/. This Registered Report has been officially endorsed by Peer Community in Registered Reports: https://doi.org/10.24072/pci.rr.100801.

## 1. Background

Research by Arkes [1] demonstrated that people have an aversion to wastefulness and that they may even make choices that compromise their own self-interest to avoid waste and appearing wasteful.

As an example, imagine a scenario in which Mary visited an amusement park and had the option of buying a single ticket or a season pass. Given that she only planned to visit once that year, she chose the cheaper single ticket option, which best aligned with her economic interest at that point in time. However, later, she was unexpectedly invited to join to go to the park again in the same year, and therefore, faces a dilemma—She would have been able to visit the park for free had she spent an extra small amount to get the season pass, and so now buying the extra ticket feels to her and appears to others as wasteful. This example shows that people may consider waste and the appearance of wastefulness when they make decisions.

Arkes [1] reported three studies testing different instances of wastefulness. The first study examined overspending, in which an individual faces a dilemma of whether to spend more if it appears wasteful, much like our opening example. The second study examined the definition concerning underutilization, in which a previously purchased item has not been fully utilized. The third study made the connection between wastefulness and the classic sunk-cost effect (or, ‘escalation of commitment’), wherein withdrawing from a course of action with time, money or effort sunk costs feels wasteful and therefore avoided.

We conducted a replication and extension Registered Report of Arkes [1] with the following main goals. Our first goal was to conduct an independent close replication of a classic article demonstrating the phenomenon of avoiding the appearance of wastefulness in decision-making, following the recent growing recognition of the importance of reproducibility and replicability in psychological science (e.g. [2,3]). Our secondary goal was to build on the target’s design and add extensions to refine the target’s methods and gain further insights. We added three extensions examining: (i) whether people indicate waste as a factor impacting their decisions in these situations, (ii) a continuous measure of willingness to engage in behaviours to supplement the target’s dichotomous choice measure, and (iii) the degree to which participants perceive the different options as wasteful, serving as the missing manipulation check.

We begin by introducing the chosen article for replication—Arkes [1]. We then discuss our motivations for the current replication and review the article by Arkes [1] and the theory and hypotheses. Finally, we outline our chosen studies for replication from the target article, the target’s experimental design and our adaptations and extensions.

### 1.1. Choice of article for replication: Arkes (1996)

We chose Arkes [1] based on several factors: its academic and practical impact, the potential for improvements in methodology and extensions to gain additional insights and the absence of direct replications.

The article has had a significant impact on scholarly research in the areas of judgement and decision-making and behavioural economics. At the time of writing (September 2024), the article had 335 Google Scholar citations. In addition, Arkes’ [1] work on waste aversion has important practical implications, such as in the domains of consumer decision-making [4] and in the links to other classic phenomena such as the sunk-cost effect [5] and ‘less is more’ (e.g. [6]).

The studies had very small samples (studies 1, 2 and 3 had 48, 55 and 55 participants, respectively) and the findings were reported briefly and were mostly descriptive, with one of the studies not reporting any statistical tests. This is understandable given the decision-making literature at the time, yet pointing to the need to revisit and reproduce the procedures, analyses and findings, to allow others to assess and better build on these findings. To our knowledge, there are currently no published direct independent replications of this article.

Going beyond the direct replication, the straightforward design of the studies allowed for the inclusion of extensions, such as: (i) a needed manipulation check of the perceived level of wastefulness, (ii) a quantitative analysis of the reasons underlying people’s decision-making (replacing the qualitative approach in the target article), and (iii) a continuous preference scale to complement the original forced choice measure.

### 1.2. Replicating studies 1, 2 and 3

We aimed to replicate all three studies reported by Arkes [1]. We summarized the set-up of studies 1, 2 and 3, along with the corresponding hypotheses and findings from the target article in tables 1 and 2. We provided more detailed study design tables of the experiments in tables 3–5.

**Table 1.** Arkes [1] studies 1, 2 and 3: Summary of hypotheses and findings.

Study	Scenario		Hypothesis	Findings of the target article
1	Constrained to specific movie nights: Mr. Munn (three-movie bundle possible; bought single tickets); Mr. Fry (no movie bundle possible; bought single tickets). Mr. Munn chose individual tickets ($5 each) over a discounted three-pack ($12). After two movies, a schedule change introduces a new movie in which both are interested.		People perceive those who rejected an opportunity to save on a purchase as less willing to make the purchase.	70.8% of participants (34 out of 48) thought Mr. Munn will be less likely to purchase the third ticket after initially forgoing the $12 three-pack.
2	Tax program bought in the previous year becomes worthless due to changes in tax laws; participants need to decide whether to purchase a new program. In one condition, participants receive a rebate for the old program, those in the other condition do not. Both spend the same amount.		Having purchased an old program, people are more willing to purchase a new program when they can receive a rebate on the old purchase compared with when they cannot.	Purchased new program waste (no rebate) condition: 11.5% (3 of 26). No-waste (rebate) condition: 37.9% (11 of 29). *χ*²(1) = 5.03, *p* < 0.05.
3	With the tent project 90% complete (cost $40 000), a competitor offers a better alternative. Decision: invest more to complete the project or abandon it for $5000. In one condition, the abandoned project is sold for scrap (no utilization), in another, it is sold to another roofer who can utilize it.		People are more likely to escalate commitment to a losing project when abandoning the project means forgoing any utility that goes beyond mere parts.	Escalated commitment: no utility: 23 of 26 (88%) utility: 19 of 26 (66%) *χ*²(1) = 4.15, *p* < 0.05.

	Extensions	Hypothesis		
1−3	Reasons	Exploratory competing hypotheses: rational: people rate utility as the most important reason. Non-rational: people rate utility similarly or lower than other reasons. Waste: people rate waste as higher than utility. Waste-top: people rate waste as the most important reason. Interaction: we expected bigger emphasis on waste in the waste condition.		
	Willingness	There is a negative association between perceived wastefulness of a certain action and the willingness to engage in that action.		
	Perceived wastefulness (manipulation check)	Wasteful behaviours (Mr. Munn, no rebate, no utilization) are perceived as more wasteful compared with the alternative behaviours (Mr. Fry, rebate, utilization).		

**Table 2.** Arkes [1] studies 1, 2 and 3: Summary of findings.

Study 1 (*N* = 48)						
**Dependent variables**	/	**d.f**.	***p***	**Effect size**	**CIL**	**CIH**
Likelihood of purchasing the third ticket		/	<0.05	Cohen’s *h*/*w* = 0.43	0.03 0.25	0.83 1.00

**Study 2 (*****N*** **= 55)**						
**Dependent variables**	***χ*** ^**2**^	**d.f**.	***p***	**Effect size**	**CIL**	**CIH**
Choice of whether to purchase a new package	5.03	1	<0.05	Cohen’s *w* = 0.27	0.00	0.55

**Study 3 (*****N*** **= 55)**						
**Dependent variables**	***χ*** ^**2**^	**d.f.**	***p***	**Effect size**	**CIL**	**CIH**
Choice of whether to continue a failing project	4.15	1	<0.05	Cohen’s *w* = 0.23	0.00	0.52

Note. CIL = lower bounds for CIs. CIH = higher bounds of CIs. Effect sizes for all three studies and confidence intervals for studies 2 and 3 were not reported in the target article and are based on our reconstructed calculations. In study 1, first-line CIs are for Cohen’s *h* 2 × 2, and second-line CIs are for Cohen’s *w* 3 × 2. See the accompanying Rmarkdown file for details and calculations.

**Table 3.** Scenario 1 (overspending): Replication and extension experimental design (one-sample proportion).

Scenario: Mr. Munn and Mr. Fry each live in an apartment near the local movie theatre Mr. Munn can go to the movies only on Monday night. Mr. Fry can go to the movies only on Friday night. Each movie costs $10, no matter which night it is shown. Each movie generally is shown for a whole week. Since Monday night is generally a pretty ‘slow’ night at the movies, the manager of the theatre offers a package to those who go to the movies on Mondays. Although tickets are $10, the manager will sell a three-pack for $24. The three-pack can be used on any three Mondays during the next month. Mr. Munn looks over the schedule for the next month and sees only two movies he is interested in seeing. So he decided not to buy the three-pack. Instead, he pays $10 on each of the first two Mondays of the month to see a movie. Mr. Fry also pays $10 on each of the first two Fridays of the month to see a movie. Then there is a change in the schedule. One of the movies that was supposed to come that month cannot be obtained. Instead, the manager substitutes a new movie that both Mr. Munn and Mr. Fry are somewhat interested in seeing. Had Mr. Munn bought the three-pack, he could have seen this new movie without paying any more money than the extra $4 he would have needed to buy the $24 three-pack. Since he did not buy the three-pack, both Mr. Munn and Mr. Fry will have to pay $10 to see the new movie.
Dependent variables: **Likelihood of purchasing the third ticket [replication]** ‘Will one of the two men be more likely to pay to see the new movie?’ −1 = Mr. Munn (three-movie bundle possible; bought single tickets) will be more likely to pay to see the new movie 0 = they will be equally likely to pay to see the new movie 1 = Mr. Fry (no movie bundle possible; bought single tickets) will be more likely to pay to see the new movie [appearance of waste]
**Reasons for predicting the likelihood of purchasing the third ticket [Extension]** ‘To what extent did the following reasons influence your decision?’ reasons: — option chosen minimizes waste — option chosen minimizes negative emotions (regret, anger, sadness, shame, etc.) — option chosen maximizes value per money spent (benefit, enjoyment, convenience, etc.) — option chosen is more consistent with previous behaviour and decisions Scale: 0 = *did not influence at all*; 6 = *influenced very much* **Willingness for Mr. Munn and Mr. Fry purchasing the additional ticket [extension]** ‘How willing do you think Mr. Munn (three-movie bundle possible; bought single tickets) and Mr. Fry (no movie bundle possible; bought single tickets) are to purchase the additional ticket?’ Scale: 0 = *absolutely not willing*; 6 = *absolutely willing* (two questions, one for each) **Perceived wastefulness for Mr. Munn and Mr. Fry purchasing the additional ticket [extension]** ‘How wasteful do you think Mr. Munn (three-movie bundle possible; bought single tickets) would be if he purchased the additional ticket?’ ‘how wasteful do you think Mr. Fry (no movie bundle possible; bought single tickets) would be if he purchased the additional ticket?’ Scale: 0 = *not at all wasteful;* 6 = *very wasteful.*

Note. Prices in the replication were doubled to adjust for inflation between the years 1996 and 2024.

**Table 4.** Scenario 2 (underutilization): Replication and extension experimental design (between-subject).

Scenario: **2A:** It is now possible to buy computer programs that help you calculate your income taxes. Suppose that you have purchased one of the standard tax programs for $50, which is a very good price. This program does all your federal income tax calculations for you, and it even generates the forms you have to send into the Internal Revenue Service. Suppose you are very pleased with the product. Now it is one year later, and you have to pay your taxes for this new year. Since Congress always changes the tax laws every year, you have to buy a new computer program for your federal taxes. The old program you purchased is completely worthless this year. This year the computer program that calculates your federal taxes is being sold with a computer program that does your state taxes. (The package of two programs costs $160, but you can get them on sale for $100.) Since you cannot buy the programs separately, you will have to spend $100 if you want to do your taxes with a computer. Of course, you can save $100 by doing your state and federal taxes by hand without the computer programs. **2B:** Scenario 2B was identical to 2A, except that the bracketed portion of 2A was replaced with the following: The package of two programs costs $160. However, the money you spent on last year’s program is not wasted; the company that sells the programs is offering a $60 rebate to people who bought last year’s federal tax computer program. If you send in your old computer program, they will give you a $60 reduction in the $160 purchase price so that the package of two new programs will cost you only $100.	
**Waste (no rebate) (2A) condition** Spend $100 on the new tax package (appearance of waste)	**No waste (rebate) (2B) condition** Trade in the old tax program from last year and use a $60 rebate to buy the new tax package at $100
Dependent variables: **Choice of whether to purchase a new package [replication]** ’Would you be willing to spend $100 for the package of two computer programs to do your taxes?’ 1 = *yes* ; 0 = *no* **Reasons for choosing whether to purchase a new package [extension]** ‘To what extent did the following reasons influence your decision?’ Reasons: — option chosen minimizes waste — option chosen minimizes negative emotions (regret, anger, sadness, shame, etc.) — option chosen maximizes value per money spent (benefit, enjoyment, convenience, etc.) — option chosen is more consistent with previous behaviour and decisions. Scale: 0 = *did not influence at all*; 6 = *dnfluenced very much* **Willingness to purchase new package [extension]** ‘How willing are you to purchase the new package for $100?’ Scale: 0 = *absolutely not willing*; 6 = *absolutely willing* **Perceived wastefulness of purchasing a new tax package [extension]** ‘How wasteful do you think it is to purchase a new tax package of $100?’ ‘How wasteful do you think it is to not purchase a new tax package of $100?’ Scale: 0 = *not at all wasteful*; 6 = *very wasteful.*	

Note. Prices in the replication were doubled to adjust for inflation between the years 1996 and 2024. The target article had slight grammar issues such as ’As you many know’, which possibly meant ’As you may know‘, and reference to a marketing survey. We removed/adjusted those.

**Table 5.** Scenario 3 (presence of sunk cost): Replication and extension experimental design (between-subject).

Scenario: As the owner of your own company, you have used $80,000 of your company’s research funds to develop a type of plastic cloth that would be used to manufacture camping tents. This material is very light, so backpackers would find it easy to carry from one campsite to another. Furthermore, it is completely waterproof, so it could keep campers dry, no matter how hard it was raining. The best part is that the cloth cannot be punctured. It is so durable that campers can use it without fear of accidentally damaging the tent. When the project is 90% completed, another firm begins marketing a waterproof tent that is made of material that is more durable than the material you have developed. It is also apparent that their tent is much cheaper than the tent you are building, and furthermore, it is much lighter. The question is: should you invest the last 10% of your research funds to finish your tent, or should you just abandon the project?	
**IV1** **: Sell to roofer** ( **3A)** Sell the unfinished tent project for $10,000 to the roofer If you abandon the project, a roofer said that he’d buy all the cloth you’ve developed so far for $10,000. He wants to sew all the tent-sized pieces together into one big tarp. He said this would come in handy as a waterproof tarp to place over roofs after he’s taken old shingles off. If it rains before he gets the new shingles on, the exposed wood on the roof would be protected by the big tarp. Unfortunately, you cannot manufacture the cloth in this large size, and nobody wants a tarp of tent-sized pieces sewn together. The roofer can really use the cloth you’ve manufactured so far, however.	**IV1: Sell for its scrap value (3B)** Sell the unfinished tent project for its scrap value of $10,000 (appearance of waste) If you abandon the project, you could sell all the cloth you’ve developed for its scrap value, which is $10,000.
Dependent variables: **Choice of whether to continue a failing project [replication]** ‘Please check the option you prefer’: 1 = invest the last 10% of your research funds to finish your tent (you have invested $80,000) (escalation) 3A—0 = abandon the project and sell the tent material to the roofer for $10,000 (de-escalation) 3B—0 = abandon the project and sell for its scrap value of $10,000 **Reasons for choosing whether to continue the project [extension]** ‘To what extent did the following reasons influence your decision?’ Reasons: — option chosen minimizes waste — option chosen minimizes negative emotions (regret, anger, sadness, shame, etc.) — option chosen maximizes value per money spent (benefit, enjoyment, convenience, etc.) — option chosen is more consistent with previous behaviour and decisions. Scale: 0 = *did not influence at all*; 6 = *influenced very much* **Willingness to continue the project [extension]** ‘How willing are you to invest the last 10% of your research funds to finish the tent project, in which you have invested $80,000?’ Scale: 0 = *absolutely not willing;* 6 = *absolutely willing* **perceived wastefulness of abandoning the project [extension]** ‘How wasteful do you think it is to abandon the tent project?’ ‘How wasteful do you think it is to finish your project?’ Scale: 0 = *not at all wasteful*; 6 = *very wasteful.*	

Note. Prices in the replication were doubled to adjust for inflation between the years 1996 and 2024.

#### 1.2.1. Study 1: Movie package

Study 1 examined the first concept of wastefulness, namely, spending more than necessary. The hypothesis was that people tend to view those who have passed up a chance to save on a present purchase as less likely to proceed with the purchase.

Arkes [1] conducted an experiment with 48 university students. The study was centred around a hypothetical scenario involving two individuals, Mr. Munn and Mr. Fry, who had different options for attending a local movie theatre. In the scenario, Mr. Munn had the option to purchase a discounted three-pack of tickets for Monday movie nights, but chose to buy individual tickets at $5 each. Mr. Fry, who could only attend on Fridays, did not have the three-pack option and also bought individual tickets. After both men had attended two movies, a schedule change introduced a new movie that both were interested in. The catch was that seeing this new movie would cost the typical $5 for a single ticket, yet if Mr. Munn had purchased the three-pack, then he could have watched it for free without any extra cost. Participants rated which of the two they thought would be more likely to purchase the third ticket and explained their choice in one or two sentences. Their answers were then manually qualitatively coded into several categories of reasons.

The findings were that a majority of participants (70.8%, or 34 out of 48) thought that Mr. Munn would be less likely to purchase the third ticket after initially forgoing the $12 three-pack. The main reasons identified were ‘wasting or losing money’, ‘expression of negative emotions’ and ‘anticipated enjoyment of the movie is worth the price.

#### 1.2.2. Study 2: Tax program

The second study in Arkes [1] demonstrated underutilization, as an additional kind of wastefulness. The idea was that individuals who bought software that has become outdated would be more inclined to invest in buying new updated software if they perceived the new purchase to be less wasteful. For example, receiving a rebate for the previous purchase would seem less wasteful, compared with when there is no use for the old software, even when the overall costs are the same, and the actual rebate does not matter other than the perception of waste.

To test this hypothesis, 55 participants were divided into two conditions of underutilization (waste) or not. In a hypothetical scenario, they were asked to imagine that they had previously bought a computer program for their income tax calculation. However, due to an annual change in the tax laws, the old purchased program was now obsolete. They then have to decide whether to purchase a new program, priced at $50, and provide reasons for their decision. In the first condition, the old program had no trade-in value. In the second scenario, the company provided a $30 rebate for the old program, which brought down the new program’s cost to $50, identical to the cost in the first scenario.

They found that 3 out of 26 subjects (11.5%) in the waste (no rebate) condition chose to buy the program. However, 11 out of 29 subjects (37.9%) in no-waste (rebate) condition made the same purchase (difference: *χ*²(1) = 5.03, *p* < 0.05).

#### 1.2.3. Study 3: Tent project

Arkes’ [1] study 3 examined the link between perceived waste (or less utility) and the sunk-cost effect, suggesting that people are less likely to escalate commitment to a losing course of action when abandoning a project is perceived as wasteful.

A total of 55 participants read a scenario, in which they owned a company that had already invested $40 000 into a tent project that was 90% complete. However, as in classic sunk-cost effect scenarios, at that point in time, they learn that a competitor introduced a superior and cheaper alternative. Participants decided between investing more to complete the project or abandoning it for $5000. In one condition, abandoning the project means selling it for scrap (no utilization). In another condition, abandoning the project means selling it to a roofer who can utilize it beyond its mere parts, therefore appearing to have more utility and less waste.

The findings were that in the no utility condition, 23 out of 26 participants (88%) chose to continue the project and escalate their commitment to a losing course of action. In contrast, in the utility condition, only 19 out of 26 participants (66%) chose to continue the project (*χ*²(1) = 4.15, *p* < 0.05).

### 1.3. Extensions

We aimed to extend the replication study by examining the reasons, willingness and perceived wastefulness, and included extensions in the experiments’ design tables (tables 3–5).

#### 1.3.1. Reasons

We aimed to quantitatively examine the determinants underpinning individuals’ decision-making processes within the context of avoiding the appearance of wastefulness. In the original studies 1 and 2, Arkes [1] asked participants to write down their reasons for their choices. In study 1, Arkes, along with two independent raters, analysed and categorized the written rationales. They excluded two responses that opted for Mr. Munn (three-movie bundle possible; bought single tickets) being more likely to buy the extra ticket, as one subject ‘obviously misunderstood the scenario’, and the other response was incomprehensible. They then identified four categories. In study 2, Arkes did not analyse the answers because they were ‘theoretically uninformative’, without providing any examples. Nonetheless, the answers provided valuable insights into how people interpreted the scenarios and made decisions. Apart from the hypothesized reason of avoiding the appearance of wastefulness, it also helped explain if people made other choices.

Given that the coding procedure was not provided and the process involved a qualitative process with subjective ratings that may result in very different insights that would be challenging to compare with the original, we decided to instead build on the target’s design and categorization, and switch from an open qualitative design to a fixed quantitative design. This also allowed us to implement this extension in all scenarios.

For the list of rated reasons, we included the first three reasons mentioned in the original study 1. We derived the fourth reason from study 3, which examined the sunk-cost effect. This reason was added to explore the influence of past behaviour and decisions on current choices, as individuals might prefer to be consistent with their previous actions, especially when faced with sunk costs. This reason was applicable to all three studies, as each contains some form of an initial decision with a change in the situation.

According to the rational agent model in neoclassical economics, the top reason would be to maximize utility. Given that the core argument of the target article is that people have considerations of waste that sometimes conflict with maximization of utility, this means that waste, emotions, or past behaviour may be rated as higher priority than utility maximization. We therefore had competing hypotheses: The neoclassical hypothesis is that utility maximization would be the strongest reason, the target article’s hypothesis given the emphasis on waste would be that ratings of waste reason would be higher than that of utility maximization and two additional possibilities are hypotheses countering the neoclassical agent model that past behaviour or emotions would be higher than utility maximization. We planned an exploratory analysis comparing the different reasons, and expected that (if waste indeed has an impact on decisions) people would rate waste as higher than utility. We outlined the competing hypotheses in table 1.

#### 1.3.2. Willingness (to complement the forced choice)

The original studies by Arkes [1] focused on binary choices, without delving into the nuances of the decision-making process. We added an extension to examine evaluations of the described agents’ willingness towards the different choices on a continuous scale, to go beyond the forced choice to examine how participants perceive each choice, allowing for a more nuanced understanding quantifying the extent to which one option is preferred over the other.

We hypothesized that individuals are more willing to choose behaviours perceived as less wasteful. This extension was applicable to all three studies in the original research by Arkes and allowed us to examine varying degrees of willingness to avoid wasteful behaviour.

#### 1.3.3. Perceived wastefulness (needed manipulation check)

In this extension, we aimed to examine the extent to which individuals perceive wastefulness in behaviours presented in the study scenarios. Given the inherent subjectivity of the concept of wastefulness and the potential for diverse interpretations, it is crucial to ensure that the different conditions manipulating wastefulness are indeed working as intended. When reproducing these scenarios, we were concerned about a possible discrepancy between Arkes’ [1] conceptualization of the concept of wastefulness and the laypersons’ perspective of wastefulness, given that there were no pre-tests reported and no included manipulation checks. As an exploratory direction, we also were interested in the differences in the strength of the wastefulness manipulations across the different scenarios and the association between manipulation strength (as indicated by the manipulation checks) with the wastefulness avoidance effect.

### 1.4. Pre-registration and open science

We provided all materials, data and code on https://osf.io/gf8rc/. This Registered Report was submitted to *Royal Society Open Science* following peer review and recommendation for stage 2 acceptance at the *Peer Community In* (PCI) *Registered Reports’* platform. Full details of the peer review and recommendation of the paper at PCI Registered Reports may be found at the links below. After submission to the journal, the paper received no additional external peer review but was accepted on the basis of the Editor’s recommendation according to the RSOS PCI Registered Reports’ policy (https://royalsocietypublishing.org/rsos/registered-reports#PCIRR). Stage 1 recommendation and review history: https://rr.peercommunityin.org/articles/rec?id=657; https://osf.io/r7tsw/ (our frozen pre-registration version of the entire stage 1 packet: https://osf.io/8fh43/). Stage 2 recommendation and review history: Markant [7]; https://doi.org/10.24072/pci.rr.100801. All measures, manipulations and exclusions conducted for this investigation are reported, and data collection was completed before conducting the data analyses. The project was part of a large mass replications and extensions project, which received ethics approval from the University of Hong Kong (#EA220438). This Registered Report was written using the Registered Report template by Feldman [8].

## 2. Method

### 2.1. Power and sensitivity analyses

We calculated effect sizes (ES) and power based on the statistics reported in the target article. We then conducted a power analysis based on the smallest effect size of interest. Effect size and power were all calculated with the help of a guide by Jané *et al*. [9] and R (version 4.3.2 [10]) using package ‘pwr’ (version 1.3-0 [11]) and G*Power 3.1 [12] for the factors that the authors found support for in the target article (flagged as significant results).

We calculated the effects in study 1 to be Cohen’s *h* = 0.43 [0.03, 0.83] with a required sample size of 70, effect in study 2 to be Cohen’s *w* = 0.27 [0.00, 0.55] with a required sample size of 179 and effect in study 3 as Cohen’s *w* = 0.23 [0.00, 0.52], with a required sample size of 240. We aimed for 95% power with an alpha of 0.05 across all analyses.

Rounding up to the highest minimum sample size required for three studies, we concluded that the minimum required sample size was 240 participants in total. We provided more information regarding these calculations in an accompanying Rmarkdown file on the OSF and ‘Power analysis of the target article effects to assess required sample for replication’ subsection of the electronic supplementary materials.

Given the likelihood that the target article’s effects are overestimated, we used the ‘small-telescope’ approach [13], aiming for enough power to detect effects much weaker than those reported by the original study (*d*_33%_) with the general rule of thumb to multiply the estimated required sample of 240 by 2.5, even if meant for other designs. This resulted in a sample of 600, more than 10 times bigger than the largest sample in the target article, and more than 3 times bigger than all the samples combined. As a reminder, to allow for an easy comparison, the target article study 1 had 48 participants, and studies 2 and 3 had 55 participants.

Accounting for our integrated design and allowing for the potential of additional analyses, we aimed for a larger total sample of 660 participants. A sensitivity analysis using GPower [12] indicated that a sample of 600 would allow the detection of effect size Cohen’s *h* = 0.20 for *z* test for study 1 (95% power, alpha = 5%, two-tail) and effect size Cohen’s *w* = 0.15 for chi-square tests for both studies 2 and 3 (both 95% power, alpha = 5%, one-tail), which are effects much weaker than any of the effects reported in the original.

### 2.2. Participants

We recruited a total of 659 US Americans on Prolific (*M*_age_ = 45.47, s.d. = 14.13; 335 females, 310 males, 14 others or did not disclose). We summarized a comparison of the target article samples and the replication samples in table 6. We targeted US Americans using Prolific’s filters. We restricted the location to the US using ‘standard sample’, we set it to ‘Nationality: United States’, ‘Country of birth: United States’, ‘Place of most time spent before turning 18: United States’, ‘Minimum Approval Rate: 95, Maximum Approval Rate: 100’, ‘Minimum Submissions: 100, Maximum Submissions: 10000’.

**Table 6.** Differences and similarities between the original study and replication

	Arkes [1] study 1	Arkes [1] study 2	Arkes [1] study 3	US Americans on **P**rolific
Sample size	48	55	55	659
Geographic origin	US	US	US	US American
Gender	not reported	not reported	not reported	310 males, 335 females, 12 other, 2 rather not disclose
Median age (years)	not reported	not reported	not reported	44
Average age (years)	not reported	not reported	not reported	45.47
S.d. age (years)	not reported	not reported	not reported	14.13
Age range (years)	not reported	approximately 41 (75%) participants aged between 30 and 40; approximately 7 (12.5%) participants aged between 18 and 22 (typical undergraduate age)	not reported	19−82
Medium (location)	not reported	not reported	not reported	computer (online)
Compensation	not reported	not reported	not reported	nominal payment
Year	1996	1996	1996	2024

### 2.3. Design: Replication and extension

We summarized the experimental designs in tables 3–5. Studies 1, 2 and 3 in the target article were conducted separately with independent samples. We ran the three scenarios together in a single unified data collection—participants completed all three scenarios in random order. The display of scenarios and conditions was counterbalanced using the randomizer ‘evenly present’ function in Qualtrics.

Scenario 1 had no manipulations, and all participants answered the same questions. In scenarios 2 and 3, participants were randomly assigned to either the wastefulness or the control condition. All three scenarios were presented in random order, and participants were randomly and evenly assigned to different conditions in scenarios 2 and 3. This unified design combining replications of several studies into a singular data collection was previously tested successfully in many of the replications and extensions conducted by our team (e.g. [14–17]) and is especially powerful in addressing concerns about the target sample (e.g. naivety and attentiveness) when some studies replicate successfully whereas others do not, as well as in allowing for drawing inferences about links between the different studies and consistency in participants’ responding to similar decision-making paradigms. In case we fail to find support for the target article’s hypotheses, we will test for order effects (order as a moderator) and for effects for each scenario when it is displayed first.

### 2.4. Procedure

We reconstructed the target’s stimuli and adjusted them to an online Qualtrics survey based on the information provided in the article.

Participants first indicated their consent, with four questions confirming their eligibility, understanding and agreement with study terms, which they must answer with a ‘yes’ and required responses in order to proceed to the study. Three of the four questions also served as attention checks, with a randomized display order of the options (yes, no, not sure)—(i) ‘Are you able to pay close attention to the details provided and carefully answer questions that follow?’, (ii) ‘Do you understand the study outline and are willing to participate in a survey with comprehension checks?’, and (iii) ‘Are you a native English speaker born, raised, and currently located in the US?’. These were followed by writing or copy-pasting a statement indicating that they understand and agree and terms, which participants had to enter correctly in order to proceed, with as many attempts as needed. Upon completion of these steps, participants proceeded to begin the survey.

Participants then answered scenarios 1, 2 and 3, presented in random order. For scenarios 2 and 3, they were randomly assigned to one of the two experimental conditions (waste versus non-waste) and responded to the assigned conditions accordingly.

We also added two multiple-choice comprehension checks presented after the scenario description, which participants had to answer correctly before proceeding to the dependent measures. If answered incorrectly, participants are asked to re-examine their responses with as many attempts as needed until they answer correctly. This procedure was designed to signal the importance of carefully reading and comprehending the scenario and to ensure that the participants read, processed and understood the key piece of information in the scenarios. We note that this is a deviation from the target’s procedure and was meant to ensure that participants understand the crucial scenario information and know what they are rating.

On the following page, participants encountered a reminder of the scenario and then indicated their decision-making choice (replication). They then proceeded to the next page for extensions. They then again read a reminder of the scenario and indicated their choices and ratings for reasons, willingness and perceived wastefulness.

At the end of the experiment, participants answered a number of funnelling and demographics questions and were debriefed.

### 2.5. Manipulations

#### 2.5.1. Scenario 1

Scenario 1 had no manipulations and contrasted waste versus no waste in a single scenario describing two people. Participants were presented with a movie package scenario where Mr. Munn faced the appearance of wastefulness for overspending—he was offered a three-movie bundle but decided to buy two single tickets, and then faced the decision of whether to purchase the third ticket for the original price. Mr. Fry was described as having no option for a movie bundle; therefore, buying the third ticket should be less of a waste. Participants indicated which of the two was more likely (or equally likely) to purchase the third ticket. The order of choice was randomized.

#### 2.5.2. Scenario 2

Participants were randomly assigned to either waste (no rebate) or no waste (rebate) conditions. They read a scenario involving the purchase of a new tax program package, given that the program purchased the previous year had become obsolete due to alterations in tax legislation. In the underutilization condition, participants read that they could purchase the new tax program on sale for $100. While in the control condition, participants read that they could use the old program to get a $60 rebate and then purchase the new tax program for $100 ($160 minus the $60 rebate). Participants then indicated their decision on whether to purchase the new software package or not (do taxes manually).

#### 2.5.3. Scenario 3

Participants were randomly assigned to one of two conditions. Participants read a sunk-cost scenario of a failing tent project and faced a choice of whether to proceed with the project or to abandon and sell it. In the no-waste (‘Sell to roofer’) condition, participants sell the project to a roofer who can utilize the remaining tent material. In the waste (‘Sell for its scrap value’) condition, participants sell the material for scrap value, which would appear wasteful. The selling price was the same in both conditions, with the only difference being whether the materials served a purpose beyond scrap value.

#### 2.6. Comprehension checks

We added two multiple-choice questions for each scenario as comprehension checks to ensure participants understood the scenario content. One question was about the general scenario context, and the second was about the manipulation. Participants had to answer these questions correctly before proceeding to the next stage to answer the dependent measures.

In scenario 1, we asked—‘Which is true for Mr. Munn?’ and ‘Which is true for Mr. Fry?’, with the following options: (i) ‘Goes to the movies on Mondays, was offered a three-movie pack for $24, and has already watched two movies ($10 each, $20 overall)’; (ii) ‘Goes to the movies on Fridays, was not offered a three-movie pack, and has already watched two movies ($10 each, $20 overall)’; (iii) ‘Goes to the movies on Mondays, was not offered a three-movie pack for $24, and has already watched two movies ($10 each, $20 overall)’; and (iv) ‘Goes to the movies on Fridays, was offered a three-movie pack, and has already watched two movies ($10 each, $20 overall)’.

In scenario 2, we asked—‘How much did you originally pay for the tax program you are no longer able to use?’ ($0, $50, $100, $160), and ‘What happens to the old tax program you bought?’ (‘You can get a full refund for it’; ‘You can trade it in for a discount on the new package’; ‘It becomes completely useless’; ‘You can still use it for federal tax this year’).

In scenario 3, we asked—‘What makes the tent developed by the other firm more competitive?’ (‘It’s more waterproof’; ‘It’s easier to carry’; ‘It’s cheaper and lighter’; ‘It’s more customizable’), and ‘What would happen if you abandon the project?’ (‘It would become useless and have no value’; ‘You could sell it in smaller pieces for various applications’; ‘You could sell it to the roofer for his tarp project’; ‘You could sell it all as scrap for $5000’).

### 2.7. Measures

We detailed the measures of the replications and extensions for each condition in tables 3–5.

#### 2.7.1. Replication: Choice

In scenario 1, participants indicated which of the two described actors representing waste versus no waste is more likely to make a purchase (−1 = *Waste*, 0 = *Equally likely*, 1 = *No waste*; participants do not see assigned value). In scenarios 2 and 3, participants indicated in their assigned conditions (representing either waste or no/less waste) whether they would make a purchase (scenario 2; 1 = *Purchase*, 0 = *Not purchase*) or continue the project with a further investment (1 = *Continue*, 0 = *Sell*).

#### 2.7.2. Extensions

##### 2.7.2.1. Reasons

Instead of the qualitative open question used in the target article, we implemented quantitative continuous measures of specific reasons deduced in the target article to measure participants’ reasons for their choices. Participants indicated the extent to which the listed reasons influenced their decision-making (0 = *Not at all*; 6 = *Absolutely*). We provided more detailed explanations about the reasons extension in the ‘Explanations for reasons extension’ section in the electronic supplementary material.

##### 2.7.2.2. Willingness

We included the degree of willingness towards behaviours perceived as wasteful or not. We utilized a Likert scale ranging from 0 (*Absolutely not willing*) to 6 (*Absolutely willing*) to gauge this measurement.

##### 2.7.2.3. Perceived wastefulness (manipulation check)

We incorporated the perception of wastefulness (0 = *Not at all wasteful*; 6 = *Very wasteful*) to assess whether the manipulation of instances of waste was indeed perceived as more wasteful and to allow a comparison of the degree of wastefulness across the scenarios.

### 2.8. Deviations

We made a few adjustments with reference to the original study design and summarized those in table 7.

**Table 7.** Replication and extension adjustments to the target article’s methods and design

Scenario	Factor	Original	Replication	Reason(s) for change
1−3	Sample characteristics	*N*_*1*_ = 48; *N*_2_ = 55; *N*_3_ = 55 University students and employees.	*N* = 659 Online recruited via Prolific.	Accounting for the possibility of underestimated effects, the unified design and multiple analyses with three extensions. More diversified sample.
	Procedure	No comprehension and manipulation check.	Comprehension checks after reading each assigned scenario. Added perceived wastefulness manipulation check extension.	Ensuring participants read and understood the scenario. Assessing manipulations are working as intended, and allowing for a comparison of wastefulness across scenarios.
		Three studies were conducted separately.	Three scenarios were combined into a unified design in a single data collection, presented in random order.	To address possible order effects. Allow comparisons and examine consistency across the different scenarios as an exploratory direction.
	Study design	Dollar amount: Study 1: $5 for each ticket and $12 for three-pack. Study 2: old tax programs for $25, new package original price of $80, on sale for $50 and $30 rebate. Study 3: used $40,000 in research funds, sell to roofer/for scrap value of $5000.	Adjusted to double the dollar amounts. Scenario 1: $10 for each ticket and $24 for three-pack. Scenario 2: old tax programs for $50, new package original price of $160, on sale for $100 and $60 dollar rebate Scenario 3: used $80 000 in research funds, sell to roofer/for scrap value of $10 000.	Accounting for inflation rate since 1990s.
1−2	Question format	Text input: ’Please write a sentence or two explaining your answer’.	Adjusted text input to a multiple-choice questions extension with continuous ratings for several fixed reasons.	Allowing for a quantitative analysis and a comparison between the reasons.

### 2.9. Evaluation criteria for replication findings

We aimed to compare the replication effects with the original effects in the target article using the criteria set by LeBel *et al*. [18].

We pre-registered our overall strategy to conclude a successful replication if all three scenarios showed a signal in the same direction as the target article, a failed replication if no scenario showed a signal in the same direction, and mixed findings if only one or two of the scenarios showed a signal in the same direction.

### 2.10. Replication closeness evaluation

We provided details on the classification of the replications using the criteria by LeBel *et al*. [19] criteria in table 8. We summarized the replication as a ‘close’ replication.

**Table 8.** Classification of the replication, based on LeBel *et al*. [19].

Design facet	Replication	Details of deviation
Effect/hypothesis	Same	
IV construct	Same	
DV construct	Same	
IV operationalization	Similar	Added comprehension checks for validation.
DV operationalization	Similar	
IV stimuli	Same	
DV stimuli	Different	Dollar amounts adjusted for inflation. Reasons presented as multiple choices instead of text input
Procedural details	Different	Three scenarios were randomly assigned and read a warning pledge before the test Added extensions.
Physical settings	Different	Online questionnaire
Contextual variables	Different/unknown	Little is known about the context. Different time and procedure.
Population (e.g. age)	Similar/different	US Americans in both. Recruited online on Prolific, a more diverse sample.
Replication classification	Close replication	

### 2.11. Data analysis strategy (pre-registered)

We performed the analyses using R (version: 4.3.2) with packages ‘jmv’ [20], ‘tidyverse’ (Wickham *et al.,* [21]) and "ggstatsplot" [22].

#### 2.11.1. Replication

To mirror the target article’s analyses, we first ran a chi-square test for scenario 2 to test the hypothesis that having purchased an old program, people are more willing to purchase a new program when they can repurpose the old purchase than when they cannot. We also ran a chi-square test for scenario 3 to test the hypothesis that people are less likely to escalate commitment to a losing course of action when the abandoned project appears to have more utility beyond mere parts.

We addressed the issue of scenario 1 being descriptive and lacking statistical tests. We aimed to extend the target article’s analyses by running a chi-square supplemented by a one-sample proportion test and an additional analysis treating the three choices as a continuous scale. The hypothesis for scenario 1 was that people perceive those who rejected an opportunity to save on a current purchase as less willing to make the purchase.

2.11.2. Extensions

For the reasons extension, in scenario 1, we ran a repeated ANOVA to compare the four reasons. In scenarios 2 and 3, we ran a mixed ANOVA with the four reasons as the within factor and the two waste conditions as the between factor.

We ran paired *t*-tests for willingness and perceived wastefulness measures for scenario 1, given the single scenario with two questions for each of these measures. We ran Welch independent samples *t*-tests for willingness and mixed ANOVA for perceived wastefulness in scenarios 2 and 3, with the rating of wastefulness of the two options as the repeated measure and waste as the between factor.

#### 2.11.3. Outliers and exclusions

We did not classify outliers in this study. All data from participants who successfully completed the survey were included.

#### 2.11.4. Order effects

One deviation from the target article was that all participants completed all scenarios in random order. We considered this to be a more robust design with many advantages, yet one disadvantage is that answers to one scenario may bias participants’ answers to the following scenarios.

We, therefore, pre-registered that if we failed to find support for our hypotheses, we would examine order as a moderator, meaning that we would run the analyses first with the unsupported study displayed first and then with the unsupported study not displayed first, and report the differences between the two, and examine whether the confidence intervals of the effect overlap. To compensate for multiple comparisons and the increased likelihood of capitalizing on chance, we set the alpha for the additional analyses to a stricter 0.005.

#### 2.11.5. Bayesian and likelihood analyses

We pre-registered that in case we failed to find support for the hypothesis for any of the scenarios, we would run a complementary Bayesian analysis for that scenario using a prior of 0.707 and report likelihood ratio tests to quantify support for the null.

## 3. Results

**Figure 1.**
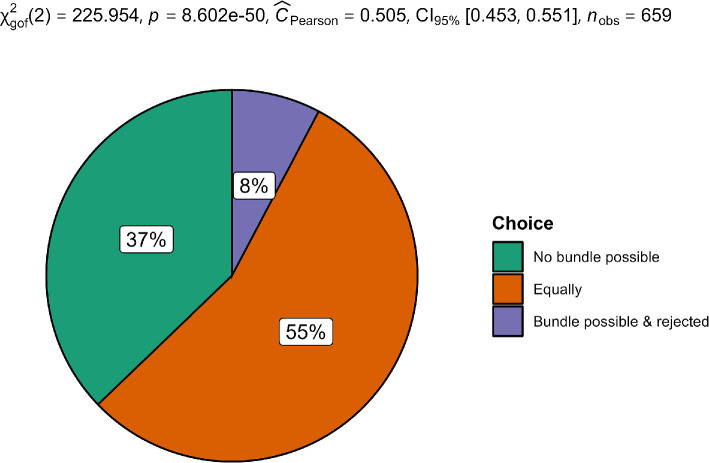
Scenario 1 (movie package): Comparison of participant choice.

**Figure 2.**
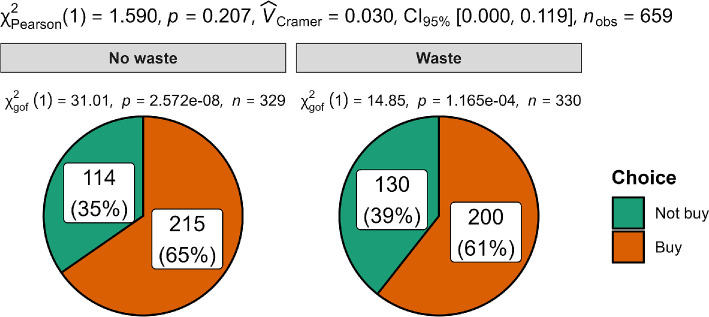
Scenario 2 (tax program): Comparison of participant choice.

**Figure 3.**
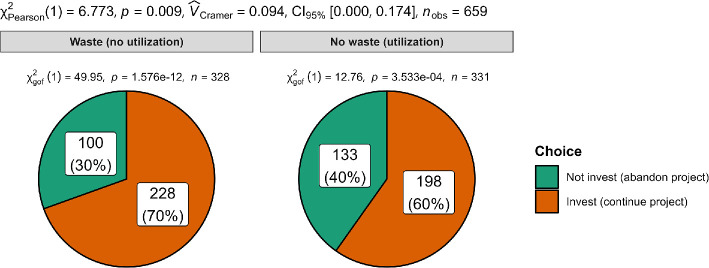
Scenario 3: Comparison of participant choice in no-waste (sell to roofer) versus waste (sell for its scrap) conditions.

We summarized the descriptives in table 9 and statistical tests in table 10, with plots in figures 1–3. Plots were created using ggstatsplot [22] and JAMOVI [23].

**Table 9.** Scenarios 1, 2 and 3: Replication descriptive statistics.

Scenario	Conditions			
**Scenario 1 movie package**	**(single scenario, no manipulation)**			
−1 = Mr. Munn (three-movie bundle possible; bought single tickets) will be more likely to pay to see the new movie 0 = They will be equally likely to pay to see the new movie 1 = Mr. Fry (no movie bundle possible; bought single tickets) will be more likely to pay to see the new movie (appearance of waste)	Mr. Fry		Equally	Mr. Munn
	*n* = 245 (37%)		*n* = 363 (55%)	*n* = 51 (8%)
**Likelihood of purchasing the third ticket**	*M* = 0.29 s.d. = 0.60			
**Scenario 2 tax program**	**Waste (no rebate) (2A)**		**No waste (rebate) (2B)**	
	*n* = 330		*n* = 329	
	Yes	No	Yes	No
Choice of whether to purchase a new package	*n* = 200 (61%)	*n* = 130 (39%)	*n* = 215 (65%)	*n* = 114 (35%)
1 = *yes* ; 0 = *no*	*M =* 0.61 s.d. *=* 0.49		*M =* 0.65 s.d. *=* 0.48	
**Scenario 3 tent project**	**Sell to roofer (3A)**		**Sell for its scrap value (3B)**	
	*n* = 331		*n* = 328	
	Finish	Abandon	Finish	Abandon
**Choice of whether to continue a failing project**	*n* = 198 (60%)	*n* = 133 (40%)	*n* = 228 (70%)	*n* = 100 (30%)
3A/B: 1 = invest the last 10% of your research funds to finish your tent (you have invested $80 000) (escalation) 3A: 0 = abandon the project and sell the tent material to the roofer for $10,000 (de-escalation) 3B: 0 = abandon the project and sell for its scrap value of $10 000	*M =* 0.60 s.d. *=* 0.49		*M =* 0.70 s.d. *=* 0.46	

Note. *N* = 600. The numbers denote the count of participants selecting each option, and percentages are shown in parentheses. *‘n*’ indicates sample size for that condition. ’M’ indicates mean. ‘s.d.’ indicates standard deviation.

**Table 10.** Scenarios 1, 2 and 3: Summary of statistical tests, effects and evaluation of current study.

		Replication				Target article			
	**scenario**	**d.f**	***χ*** **²**	***p***	**Cohen’s** ***h*** **and CI**	**d.f**.	***χ*** **²**	***p***	**Cohen’s** ***h*** **and CI**	**Interpretation**
1	Movie package (overspending) — Mr. Munn (three-movie bundle possible; bought single tickets) — Mr. Fry (no movie bundle possible; bought single tickets)	2	225.95	<0.001	0.26 [0.10, 0.42]	2	/	*p* < 0.05	0.43 [0.03, 0.83]	signal, inconsistent, weaker
	**Scenario**	**d.f**.	***χ*** **²**	***p***	**Cohen’s** ***w*** **and CI**	**d.f**.	***χ*** **²**	***p***	**Cohen’s** ***w*** **and CI**	**Interpretation**
2	Tax program (underutilization) — waste (no rebate) (2A) — no waste (rebate) (2B)	1	1.59	0.207	0.03 [0.00, 0.12]	1	5.03	*p* < 0.05	0.27 [0.00, 0.55]	no-signal, inconsistent
3	Tent project (presence of sunk cost) — sell to roofer (3A) — sell for its scrap value (3B)	1	6.77	0.009	0.09 [0.00, 0.17]	1	4.15	*p* < 0.05	0.23 [0.00, 0.52]	signal, inconsistent, weaker

Note. CI = 95% confidence intervals. The interpretation of the outcome was based on LeBel *et al*. [18].

### 3.1. Replication

#### 3.1.1. Scenario 1 (overspending): Movie package

We hypothesized that people perceive those who passed up a chance for a discount as less willing to make the purchase. We conducted a one-proportion test among the three choices and found support for the hypothesis that people think Mr. Fry (no movie bundle possible; bought single tickets; count = 245, proportion = 37%) is more likely to purchase the third ticket than Mr. Munn (three-movie bundle possible; bought single tickets; count = 51, proportion = 8%), *χ²*(2, *N* = 659) = 225.95, *p* < 0.001, *h* = −0.26, 95% CI [−0.42, −0.10]. We provided a summary plot in figure 1. A one-sample *t*‐test when treating the choice as a continuous scale showed similar results (*t*(658) = −12.54, *p* < 0.001, *d* = −0.49 [−0.57, −0.41]).

#### 3.1.2. Scenario 2 (underutilization): Tax program

We hypothesized that once they have purchased an old program, people are more willing to purchase a new program when they can get a rebate on their previous purchase than if no rebate is offered. We conducted a chi-square test between two conditions, namely the ‘Waste (no rebate)’ condition and ‘No waste (rebate)’ condition, and failed to find a significant difference in people’s level of willingness to buy a new program in ‘Waste (no rebate)’ compared with ‘No waste (rebate)’ condition (*χ²*(1, *N* = 659) = 1.59, *p* = 0.207, *w* = 0.03, 95% CI [0.00, 0.12]), despite a slightly more proportion of people in ‘No waste (rebate)’ (count = 215, proportion = 65%) than ‘Waste (no rebate)’ condition (count = 200, proportion = 61%) chose to buy a new program. We provided a summary plot in figure 2.

##### 3.1.2.1. Bayesian analysis and likelihood ratio tests

We conducted a Bayesian analysis of independence for scenario 2 (tax program) using a Poisson model with a prior concentration of 1. The Bayes factor (BF01) quantifying support for the null hypothesis over the alternative hypothesis was 2.42, which is commonly considered merely anecdotal evidence in favour of the null hypothesis. We also conducted a likelihood ratio test resulting in *G*(1) = 1.59, *p* = 0.207, commonly categorized as weak to moderate evidence in favour of the null.

### 3.1.3. Scenario 3 (sunk cost): Tent project

We hypothesized that people are more likely to escalate commitment to a failing project when discontinuing it means forgoing any utility that goes beyond mere parts. We conducted a chi-square test comparing between ‘No waste (utilization)’ condition and the ‘Waste (no utilization)’ condition and found support for the hypothesis that people in the ‘Waste (no utilization)’ condition (count = 228, proportion = 70%) are more likely than ‘No waste (utilization)’ condition (count = 198, proportion = 60%) in choosing to proceed with the project, *χ²*(1, *N* = 659) = 6.77, *p* = 0.009; *w* = 0.09, 95% CI [0.00, 0.17]. We provided a summary plot in figure 3.

### 3.2. Extensions

#### 3.2.1. Reasons

We tested four competing exploratory hypotheses regarding the reasons: the neoclassical hypothesis, which posits that utility maximization would be rated highest; the hypothesis from the target article, which suggested that the emphasis on avoiding waste would be rated higher than utility maximization; and two alternative hypotheses proposing that either past behaviour or the avoidance of negative emotions would be rated higher than utility maximization (table 1). We summarized descriptives in table 11 and the statistical test findings in table 12.

**Table 11.** Scenarios 1, 2 and 3: descriptive statistics for reasons extension.

Scenario condition	Minimizes waste	Minimizes negative emotions	Maximizes value per money spent	More consistent with previous behaviour and decisions	Overall
1	*M =* 2.36 s.d. = 1.93	*M =* 2.97 s.d. *=* 1.95	*M =* 3.52 s.d. *=* 1.81	*M =* 3.97 s.d. *=* 1.69	*n* = 659 *M =* 3.20 s.d. *=* 1.94
2a	*M =* 2.84 s.d. = 2.15	*M =* 3.19 s.d. *=* 1.99	*M =* 4.52 s.d. *=* 1.49	*M =* 3.76 *s.d. =* 1.73	*n* = 330 *M =* 3.58 s.d. *=* 1.96
2b	*M =* 3.24 s.d. = 1.97	*M =* 3.20 s.d. *=* 1.96	*M =* 4.46 s.d. *=* 1.42	*M =* 3.76 s.d. *=* 1.76	*n* = 329 *M =* 3.67 s.d. *=* 1.86
3a	*M =* 4.06 s.d. = 1.75	*M =* 3.37 s.d. *=* 1.71	*M =* 3.85 s.d. *=* 1.61	*M =* 3.30 s.d. *=* 1.79	*n* = 331 *M =* 3.64 s.d. *=* 1.74
3b	*M =* 4.08 s.d. = 1.72	*M =* 3.44 s.d. *=* 1.84	*M =* 4.03 s.d. *=* 1.47	*M =* 3.62 s.d. *=* 1.73	*n* = 328 *M =* 3.79 s.d. *=* 1.71

**Table 12.** Scenarios 1–3 reasons extension: Summary of statistical tests.

Scenario	Statistical test	d.f.	*F*	*p*
1	ANOVA	(3, 1974)	113.08	<0.001
2	mixed ANOVA			
	reasons (within)	(2.79, 1834.89)	100.50	<0.001
	waste condition (between)	(1, 657)	1.12	0.290
	reasons:waste interaction	(2.79, 1834.89)	2.64	0.052
3	mixed ANOVA			
	reasons (within)	(2.91, 1911.78)	32.96	<0.001
	waste condition (between)	(1, 657)	2.94	0.087
	reasons:waste interaction	(2.91, 1911.78)	1.30	0.273

Note. *CI* = 95% confidence intervals. The interpretation of the outcome was based on LeBel *et al*. [18].

##### 3.2.1.1. Scenario 1 (overspending): Movie package

**Figure 4.**
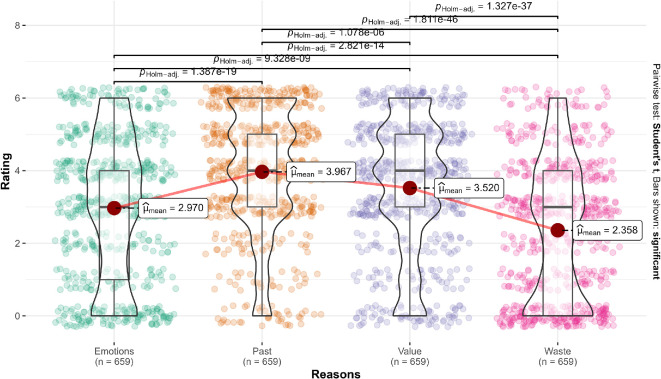
Scenario 1: Reasons extension.

We conducted a repeated ANOVA in scenario 1 and found support for differences in ratings of the importance of reasons (*F*(3, 1974) = 113.08, *p* < 0.001, *η*² = 0.10). Post hoc comparisons using the Tukey honestly significant difference (HSD) indicated differences between the four conditions. The top reason was past behaviour (*M* = 3.97, s.e.m. = 0.07; all *p*s < 0.001), followed by maximizing value (*M* = 3.52, s.e.m. = 0.07), then minimizing negative emotions (*M* = 2.97, s.e.m. = 0.08) and finally minimizing waste (*M* = 2.36, s.e.m. = 0.08). We provided a summary plot in figure 4.

To summarize, we found support for the hypothesis that people have reasons other than utility maximization that they rank as more important, though the highest was past behaviour rather than avoiding waste, which was rated the lowest. This suggests that people may have read the scenario meant to be about waste as more of a past behaviour plan and/or decision to the number of movies to watch.

##### 3.2.1.2. Scenario 2 (underutilization): Tax program

In scenario 2, we conducted a 4 (within) by 2 (between; by choice) mixed ANOVA. We found support for a main effect of reasons (*F*(2.79, 1834.89) = 100.50, *p* < 0.001, *η*² = 0.09). Post hoc test revealed that the rating of minimizing waste reason (*M* = 3.04, s.e.m. = 0.08) was lower than maximizing value (*M* = 4.49, s.e.m. = 0.06), and past behaviour (*M* = 3.76, s.e.m. = 0.07), both *p*s < 0.001. Minimizing negative emotions (*M* = 3.19, s.e.m. = 0.08) was lower than maximizing value (*M* = 4.49, s.e.m. = 0.06), and past behaviour (*M* = 3.76, s.e.m. = 0.07), both *p*s < 0.001. Maximizing value (*M* = 4.49, s.e.m. = 0.06) was higher than past behaviour (*M* = 3.76, s.e.m. = 0.07), *p* < 0.001.

**Figure 5.**
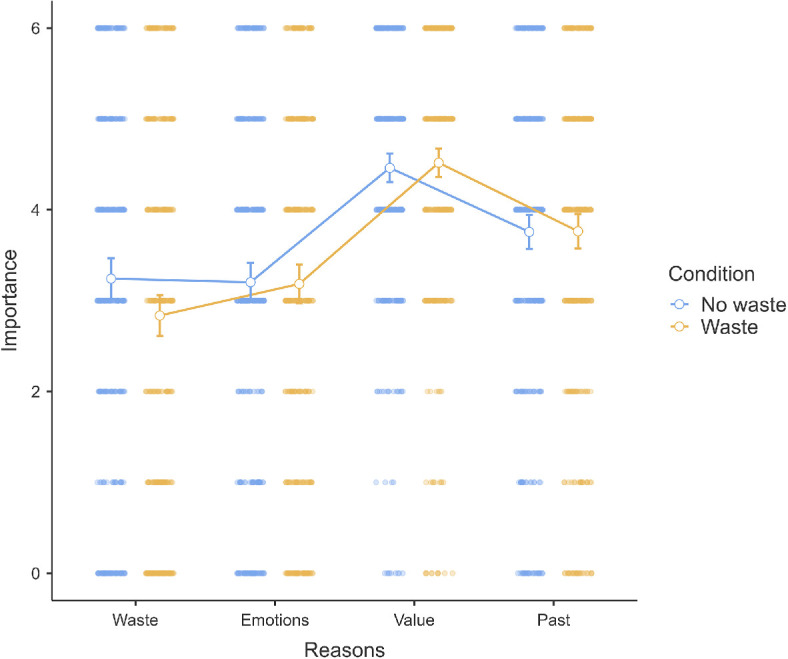
Scenario 2: Reasons extension.

We found no support for differences in ratings of the importance of reasons between the ‘Waste (no-rebate)’ and ‘No waste (rebate)’ conditions (*F*(1, 657) = 1.12, *p* = 0.290) or an interaction (*F*(2.79, 1834.89) = 2.64, *p* = 0.052). We provided a summary plot in figure 5.

Thus, we found support for the neoclassical hypothesis that people rate utility as the most important reason, with avoiding waste rated among the lowest and with no support for differences between waste and no-waste conditions. This is in line with the broader lack of support for waste as a factor in scenario 2 and noting the failed manipulation checks (§3.2.3).

##### 3.2.1.3. Scenario 3 (sunk cost): Tent project

In scenario 3, we conducted a 4 (within) by 2 (between; by choice) mixed ANOVA. We found support for a main effect of reasons (*F*(2.91, 1911.78) = 32.96, *p* < 0.001, *η*² = 0.03). The post hoc tests revealed that minimizing waste (*M* = 4.07, s.e.m. = 0.07) was higher than minimizing negative emotions (*M* = 3.40, s.e.m. = 0.07) and past behaviour (*M* = 3.46, s.e.m. = 0.07; both *p*s < 0.001). Minimizing negative emotions (*M* = 3.40, s.e.m. = 0.07) was lower than maximizing value (*M* = 3.94, s.e.m. = 0.06, *p* < 0.001). Maximizing value (*M* = 3.94, s.e.m. = 0.06) was higher than past behaviour (*M* = 3.46, s.e.m. = 0.07, *p* < 0.001) and similar to minimizing waste. Thus, we found support for minimizing waste and maximizing value as the most important reasons.

**Figure 6.**
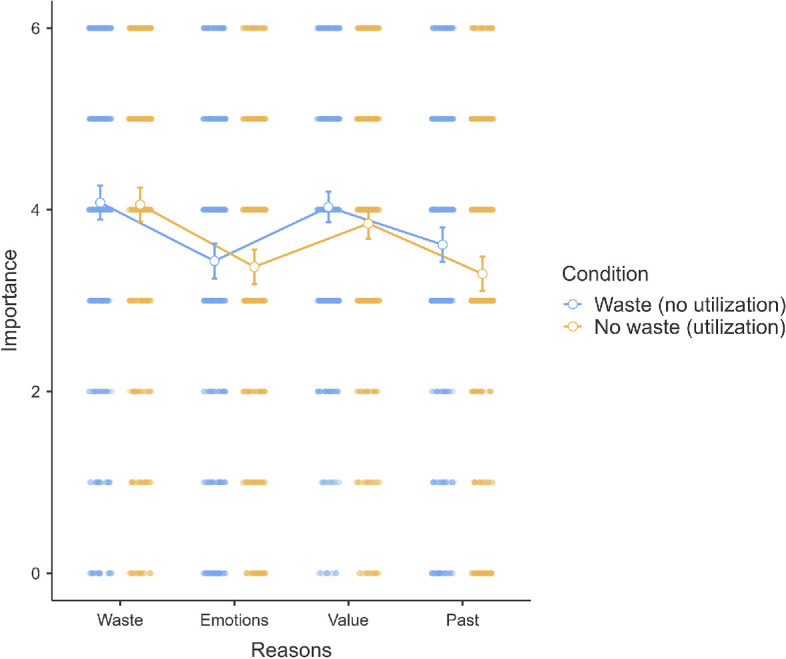
Scenario 3: Reasons extension.

We found no support for differences between the waste and no-waste conditions (*F*(1, 657) = 2.94, *p* = 0.087) or an interaction (*F*(2.91, 1911.78) = 1.30, *p* = 0.273). We provided a summary plot in figure 6.

### 3.2.2. Willingness

We hypothesized that there is a negative association between the perceived wastefulness of a certain action and the willingness to engage in that action. We summarized descriptives in table 13 and findings in table 14.

**Table 13.** Scenarios 1–3 willingness extension: Descriptive statistics.

Scenario	Waste	No waste
Scenario 1 movie package willingness to buy ticket	Mr. Munn 3.68 (1.54)	Mr. Fry 4.61 (1.42)
Scenario 2 tax program willingness to purchase program	No rebate 3.22 (2.13)	Rebate 3.49 (2.07)
Scenario 3 tent project willingness to proceed with project	Scrap value 3.90 (2.01)	Roofer 3.47 (2.08)

Note. Format = mean (standard deviation)

**Table 14.** Scenarios 1–3 willingness extension: Summary of statistical tests.

Scenario	Statistical test	*t*	d.f.	*p*	Hedges’ *g* and CI
1	Paired *t*‐test	12.46	658	<0.001	0.49 [0.40, 0.57]
2	Welch two-sample *t*‐test	1.66	656.5	0.098	0.13 [−0.02, 0.28]
3	Welch two-sample *t*‐test	2.69	656.6	0.007	0.21 [0.06, 0.36]

Note. CI = 95% confidence intervals. The interpretation of the outcome was based on LeBel *et al*. [18].

#### 3.2.2.1. Scenario 1 (overspending): Movie package

**Figure 7.**
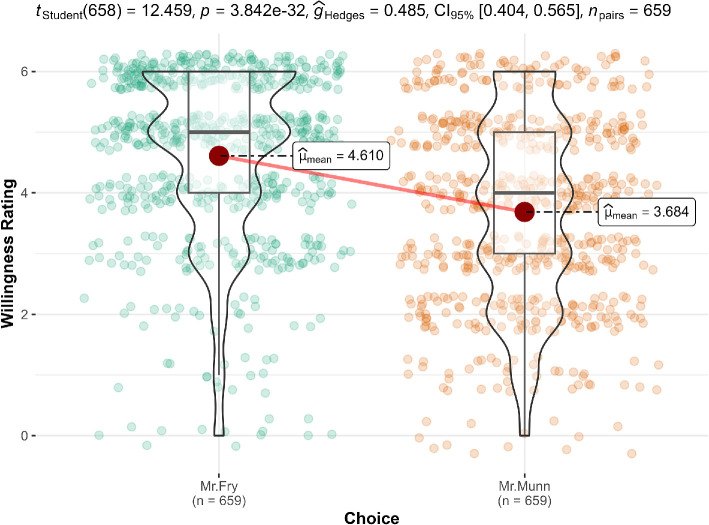
Scenario 1: Willingness extension.

We conducted a paired *t*‐test in scenario 1 and found support for the hypothesis that participants think Mr. Fry (no movie bundle possible; bought single tickets; *M* = 4.61, s.d. = 1.42) is more willing to purchase the third ticket than Mr. Munn (three-movie bundle possible; bought single tickets; *M* = 3.68, s.d. = 1.54; *t*(658) = 12.46, *p* < 0.001, *g* = 0.49, 95% CI [0.40, 0.57]). We provided a summary plot in figure 7.

#### 3.2.2.2. Scenario 2 (underutilization): Tax program

**Figure 8.**
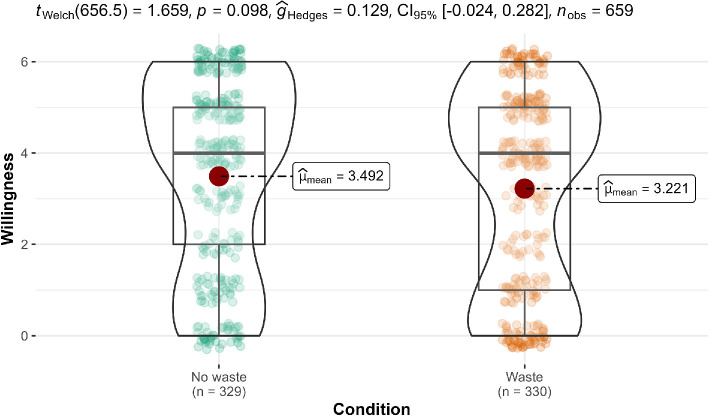
Scenario 2: Willingness extension.

We conducted a two-sample Welch’s *t*‐test in scenario 2 and found no support for the hypothesis that participants in the no-waste condition (*M* = 3.49, s.d. = 2.07) were more willing to purchase the new tax program than those in the waste condition (*M* = 3.22, s.d. = 2.13; *t*(656.5) = 1.66, *p* = 0.098, *g* = 0.13, 95% CI [−0.02, 0.28]). We provided a summary plot in figure 8.

#### 3.2.2.3. Scenario 3 (sunk cost): Tent project

**Figure 9.**
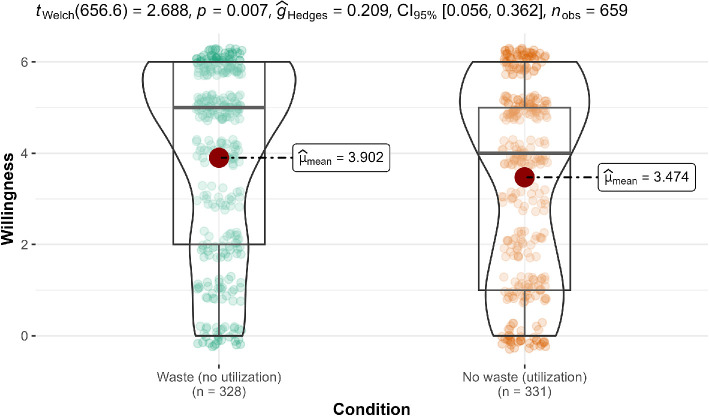
Scenario 3: Willingness extension.

We conducted a two-sample Welch’s *t*‐test in scenario 3 and found support for the hypothesis that people in the waste condition (*M* = 3.90, s.d. = 2.01) were more willing to continue the tent project compared with people in the no waste condition (*M* = 3.47, s.d. = 2.08; *t*(656.6) = 2.69, *p* = 0.007, *g* = 0.21, 95% CI [0.06, 0.36]). We provided a summary plot in figure 9.

### 3.2.3. Perceived wastefulness (manipulation check)

Perceived wastefulness served as a manipulation check that was missing from that target article, to ensure that wasteful behaviours (scenario 1’s Mr. Munn, scenario 2’s no rebate condition and scenario 3’s no utilization condition) would be perceived as more wasteful compared with the alternative behaviours (scenario 1’s Mr. Fry, scenario 2’s rebate condition and scenario 3’s utilization). We summarized descriptives in table 15 and findings in table 16.

**Table 15.** Scenarios 1, 2 and 3 perceived wastefulness extension: Descriptive statistics.

Scenario	Waste	No waste
Scenario 1 movie package	Mr. Munn 2.38 (1.76)	Mr. Fry 1.18 (1.55)
Scenario 2 tax program Buy Not buy	No rebate 2.98 (2.08) 1.81 (1.76)	Rebate 2.67 (2.03) 1.94 (1.83)
Scenario 3 tent project Proceeding Abandoning	No utilization 2.37 (1.99) 4.33 (1.79)	Utilization 2.67 (2.01) 4.06 (1.87)

Note. Format = Mean (standard deviation).

**Table 16.** Scenarios 1−3 perceived wastefulness: Summary of statistical tests.

Scenario	Statistical test	d.f.	statistic	*p*
1	paired *t*‐test	658	*t* = −15.95	<0.001
2	mixed ANOVA		*F* =	
	wastefulness (within)	(1, 657)	59.29	<0.001
	waste condition (between)	(1, 657)	1.07	0.301
	wastefulness by waste	(1, 657)	3.05	0.081
3	mixed ANOVA		*F* =	
	wastefulness (within)	(1, 657)	166.11	<0.001
	waste condition (between)	(1, 657)	0.09	0.759
	wastefulness by waste	(1, 657)	4.82	0.029

Note. CI = 95% confidence intervals. The interpretation of outcome was based on LeBel *et al.* [18].

#### 3.2.3.1. Scenario 1 (overspending): Movie package

**Figure 10.**
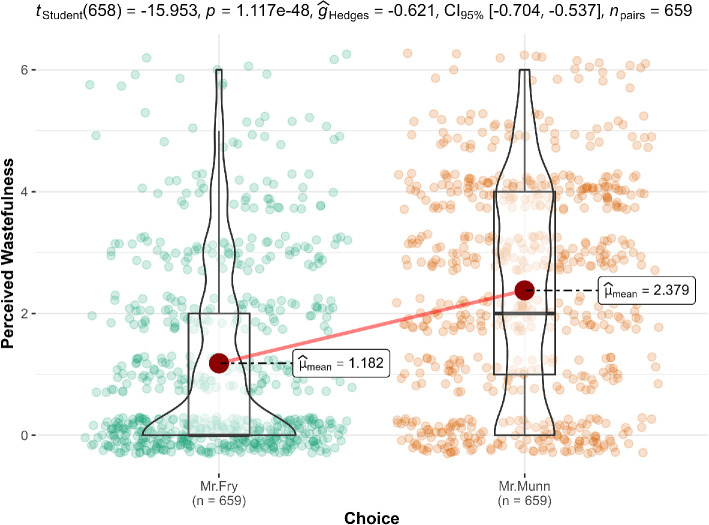
Scenario 1: Perceived wastefulness extension.

We conducted a paired *t*‐test in scenario 1 and found support for the hypothesis that people think the situation of Mr. Munn (three-movie bundle possible; bought single tickets; *M* = 2.38, s.d. = 1.76) shows more wastefulness than Mr. Fry (No movie bundle possible; bought single tickets; *M* = 1.18, s.d. = 1.55; *t*(658) = 15.95, *p* < 0.001), *g* = 0.62, 95% CI [0.54, 0.70]. We provided a summary plot in figure 10.

#### 3.2.3.2. Scenario 2 (underutilization): Tax program

**Figure 11.**
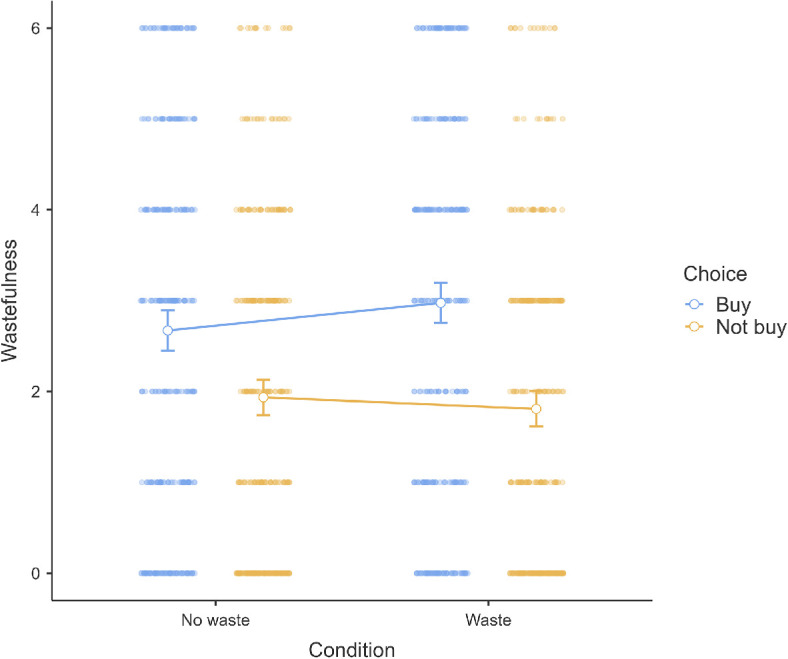
Scenario 2: Perceived wastefulness extension.

We conducted a mixed ANOVA in scenario 2 and found support for a main effect of choice (*F*(1, 657) = 59.29, *p* < 0.001, *η*² = 0.06), with buying (*M* = 2.82, s.e.m. = 0.08) higher than not buying (*M* = 1.87, s.e.m. = 0.07; *t*(657) = 7.70, *p* < 0.001). However, and more importantly, we found no support for a main effect of waste (*F*(1, 657) = 1.07, *p* = 0.301) or an interaction (*F*(1, 657) = 3.05, *p* = 0.081). We provided a summary plot in figure 11.

#### 3.2.3.3. Scenario 3 (sunk cost): Tent project

**Figure 12.**
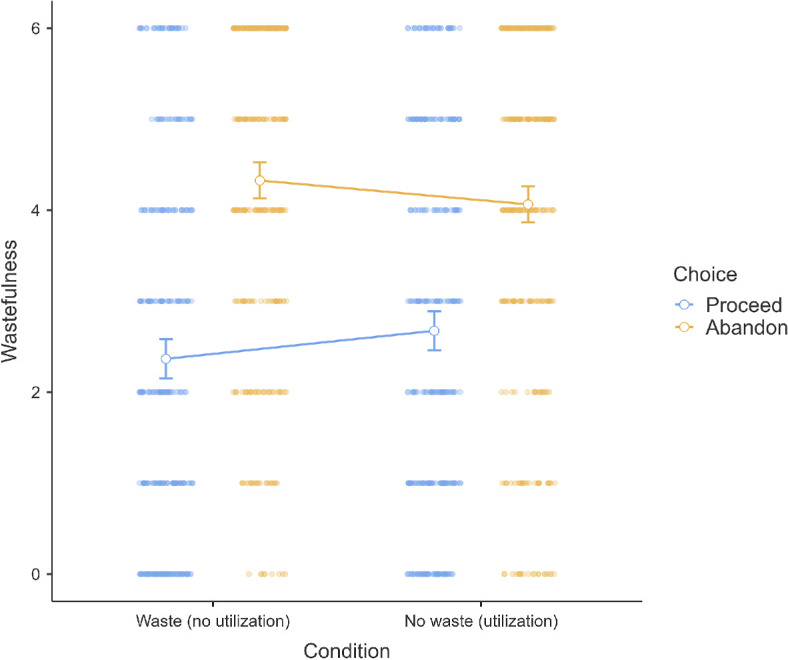
Scenario 3: Perceived wastefulness extension.

We conducted mixed ANOVA in scenario 3 and found support for a main effect of choice with differences between proceeding (*M* = 2.52, s.e.m. = 0.08) and abandoning (*M* = 4.20, s.e.m. = 0.07; *F*(1, 657) = 166.11, *p* < 0.001, *η*² = 0.16). There was no support for a main effect of waste (*F*(1, 657) = 0.09, *p* = 0.759), but with support for an interaction (*F*(1, 657) = 4.82, *p* = 0.029, *η*² = 0.01). Perceived wastefulness of abandoning (*M* = 4.33, s.e.m. = 0.10) was higher than continuing it (*M* = 2.37, s.e.m. = 0.11) under the waste condition (*t*(327) = 10.79, *p* < 0.001, *g* = 0.59, 95% CI [0.48, 0.71]), with a weaker effect in the no-waste condition (abandoning: *M* = 4.06, s.e.m. = 0.10; continuing: *M* = 2.67, s.e.m. = 0.11; *t*(330) = 7.48, *p* < 0.001, *g* = 0.41, 95% CI [0.30, 0.52]). We provided a summary plot in figure 12.

### 3.3. Additional analysis: Order effect

We did not find any indication for order effects in scenarios 1 (movie package) and 2 (tax program). In scenario 3 (tent project), the order effect analysis resulted in no support for differences between the waste conditions when scenario 3 was presented first (*χ*²(1, *N* = 225) = 0.60, *p* = 0.438, *w* = 0.05, 95% CI [0.00, 0.17]) or when scenario 3 was not the first scenario presented (*χ*²(1, *N* = 434) = 7.08, *p* = 0.008, *w* = 0.13, 95% CI [0.03, 0.22]; alpha set to 0.005), probably due to the lower power and stricter alpha threshold we set for order analysis in stage 1.^1^ As we previously noted in the peer review in stage 1,^2^ we caution against over-interpreting these order analysis findings due to the numerous ways in which order effects can be conducted, and especially given that the additional analyses severely restrict the power to detect effects. Our main focus in interpretation is the better-powered overall sample with lower alpha mirroring the analyses in the target article.

## 4. Discussion

We conducted a replication and extension Registered Report of Arkes [1], resulting in mixed findings that were only partially consistent with the original results.

### 4.1. Replication

#### 4.1.1. Scenario 1: Movie package

The results of this replication study align with Arkes’ [1] initial findings, which suggested that the perception of overspending influences economic choices.

One difference in the results was the high proportion of participants who perceived both scenarios as equally likely to lead to an additional purchase (55%; count = 363). The reasons extension offers a possible explanation, with the highest reason being past behaviour. It is possible that the decision not to purchase a bundle in this scenario was perceived as a commitment to evaluating movies per movie basis, in which case there should be fewer differences between the two conditions. A possible tweak to the scenario might be to try to disentangle waste from past behaviour and examine the interaction between the two.

#### 4.1.2. Scenario 2: Tax program

Contrary to the original study by Arkes [1], which suggested that allowing consumers to trade in an old item (thereby not wasting it) increased their likelihood of purchasing a new item, our results did not find a difference.

Several factors might account for the differences between our findings and those of the original study. The landscape of tax preparation has evolved since the original study. With free tax preparation software and services available, the perceived value of purchasing a new tax program may have diminished. This shift could reduce the attractiveness of a rebate offer, as the baseline willingness to purchase any tax software might be lower. The scenario involving tax software might not resonate as strongly with contemporary participants as it did in the past. The psychological impact of ‘wasting’ a previous version may be less pronounced as the general public becomes more accustomed to digital products that quickly become obsolete.

#### 4.1.3. Scenario 3: Tent project

The findings of our replication of experiment 3 from Arkes [1] align with the original study’s results, supporting the hypothesis that the perception of waste amplifies the sunk-cost effect, where individuals escalate their commitment to losing course of action to avoid feeling that their initial investment has been wasted.

The concept of wastefulness and its influence on decision-making highlights how cognitive biases can divert choices from rational economic behaviour. The sunk-cost effect is fuelled by the desire to justify past investments. The term ‘scrap’ probably evokes a negative connotation, reinforcing the aversion to waste, which in turn prompts individuals to continue investing in a failing venture. This pattern of decision-making aligns with what Arkes [24] identified as an ‘association-based error’, where the association of a term with negative outcomes influences decisions, even when the financial repercussions are equivalent.

### 4.2. Extension

#### 4.2.1. Reasons

We investigated the extent to which various factors influence participants’ choices across different scenarios. We identified key factors potentially impacting decision-making, including the considerations of minimizing waste, managing negative emotions, rational thinking aimed at maximizing values and the tendency to maintain consistency with past behaviour. Recognizing the relevance of each factor, we formulated four exploratory competing hypotheses to guide our analysis.

In scenario 1 (movie package), we observed that past behaviour was the most influential factor among all four considered, suggesting that people mostly are focused on the comparison of how the two agents acted before the decision. Not in line with expectations, waste was considered the least influential, and several explanations might exist. This unexpected finding might be related to different interpretations of the term ‘waste’. As one participant highlighted in the funnelling section, ‘waste’ might refer to the physical form of waste rather than the broader concept of spending more than necessary or not fully utilizing a purchased item as we originally assumed. Future research can try to identify the different meanings associated with waste and how those might relate to the interpretation of scenarios like those used in these experiments. Another possibility is that perceptions of waste are conflated or correlated with other factors that might be more salient when evaluating situations involving waste. For example, it is possible that a decision not to purchase a bundle is perceived as reflecting a decision to be more careful with spending or to commit to a certain limit in purchasing.

In scenario 2 (tax program), the reason for minimizing waste was rated as the lowest, not in line with our exploratory hypotheses for a higher emphasis on avoiding waste. Instead, participants prioritized maximization of utility, supporting the neoclassical hypothesis. The results suggest that in some decisions, the perceived necessity and functionality outweigh concerns about the waste of previous investments. We caution against over-interpreting this result given the failed manipulation checks and replication of scenario 2.

In scenario 3 (tent project), the reason for minimizing waste was rated as high as maximizing value, both rated higher than minimizing negative emotions and past behaviour. This supports the idea that decisions are influenced by factors that go beyond mere economic utility, or alternatively, that minimizing waste and maximizing utility are linked and related to one another. Future research may further investigate links between perceived waste and utility.

#### 4.2.2. Willingness (to complement the forced choice)

Our study extended the work of Arkes [1] by investigating the relationship between perceived wastefulness and willingness to engage in certain actions, allowing us to delve deeper into the subtleties of decision-making processes that may not be fully captured by binary choices. The findings for the willingness measure mirrored that of the dichotomous choice.

We expanded beyond simple binary willingness choices by utilizing a continuous scale. This approach facilitates a more nuanced understanding of willingness levels and enhances the precision of effect size estimation, allowing for deeper insights and more accurate assessments.

Furthermore, the effect size observed in scenario 1 (movie package) surpasses that in scenario 2 (tax program) and scenario 3 (tent project). Though effect sizes of different designs are tricky to compare, this may suggest that the effect is pronounced in within-subject designs compared with between-subject designs. Future research could explore designs that contrast within-subject and between-subject methodologies to investigate whether the salience of waste increases when individuals can readily compare more wasteful options against less wasteful ones.

#### 4.2.3. Perceived wastefulness (needed manipulation check)

Our study aimed to validate the operationalization of perceived wastefulness in all three scenarios, addressing concerns about the potential misalignment between theoretical conceptualizations and lay perceptions. This was essential given the absence of prior manipulation checks in the foundational research by Arkes [1]. Our findings provide insights into how these manipulations were perceived and their effectiveness in evoking a sense of wastefulness.

The results from these scenarios demonstrate that the experimental manipulations in scenarios 1 (movie package) and 3 (tent project) successfully influenced perceptions according to the design. However, the manipulation in scenario 2 (tax program) was less effective. While participants deemed the purchase wasteful, the specific manipulation involving a trade-in option did not seem to affect their perceptions of wastefulness. Given that the manipulation was not effective, it is no surprise that the waste manipulation did not work as well as expected. This shows the importance of incorporating manipulation checks to ensure that participants are processing the scenarios in the same way as intended. This would also allow us to track how the evaluation of wastefulness may differ across different scenarios and may shift in time, as well as across cultures and contexts.

### 4.3. Limitations and future directions

#### 4.3.1. Constraints on generalizability

Our findings are based on hypothetical scenarios, which, while instrumental for isolating specific variables of interest, do not capture the multifaceted nature of real-life decision-making processes. Some of our participants gave us feedback about the complexity of decisions, noting context-specific factors such as the type of tax filing (‘...there are many things to be considered in the decision-making process, so more info (e.g., tax filing; business or just regular tax filing) as this makes a HUGE dif.’). The use of simplified scenarios does not fully capture the nuanced cognitive and emotional factors driving economic behaviour in naturalistic settings. Future research may build on the scenarios we successfully replicated to attempt experimental designs with real financial decisions.

We conducted a direct replication, and we tried to stay true to the original methods best as we could. We also made all our materials, data and code available, and so in the spirit of open and large-team collaborative science, we invite researchers to utilize our replication and run similar direct replications in other contexts. We also see much value in future direct replications of other follow-up studies, conceptual replications building on our findings and aiming to identify contextual and moderating factors and a meta-analysis to provide a comprehensive summary of the existing literature on the psychology of waste.

#### 4.3.2. Unified design combining several studies in a target article

In contrast to the original article, which conducted three separate studies with underpowered samples, we integrated all three scenarios into a single data collection. This unified design ensured a sufficient sample size for detecting effects. In our replication, we found support for the findings for scenarios 1 (movie package) and 3 (tent project) yet found no support for scenario 2 (tax program). Had we only conducted a replication of scenario 2, we might have concluded no support for the phenomenon. However, by running all three scenarios together, we confirmed the validity of the overall sample while identifying specific issues with scenario 2 (tax program). That said, a unified design may also introduce bias in participants’ responses in subsequent scenarios, and we found possible indication for suggestive evidence for an order effect in scenario 3 (tent project), though we cautioned against over-interpreting this finding given that the sub-samples based on order are of lower power. Overall, we see much promise in replications employing unified designs in comprehensive replications of target articles with several studies, addressing potential concerns about the sample and attentiveness, and giving a broader more robust perspective of a phenomenon.

## 5. Conclusion

Our replication of Arkes [1] yielded mixed results. We found support for scenarios 1 (movie package) and 3 (tent project), yet failed to find support for scenario 2 (tax program), which could be explained by an added manipulation check extension where we failed to find support for the waste manipulation in scenario 2. In our extension employing a continuous willingness measure to supplement the scenarios’ dichotomous choice, we found similar results to those using the dichotomous choice. In our extension examining reasons, in the successfully replicated scenarios, we found that in scenario 1 (movie package) utility maximization was not rated as the most important, and in scenario 3 (tent project), we found that minimizing waste was rated as the most important reason.

## Data Availability

We provided all materials, data and code on [25]. Supplementary material is available online [26].

## Footnotes

^1^
Reminder. We pre-registered the following in stage 1: ‘To compensate for multiple comparisons and the increased likelihood of capitalizing on chance, we set the alpha for the additional analyses to a stricter 0.005’.

^2^
See our reply to the editor in the first revision round of the stage 1 in https://osf.io/6aksx (p. 4 #2).
